# Effects of Poloxamers as Excipients on the Physicomechanical Properties, Cellular Biocompatibility, and In Vitro Drug Release of Electrospun Polycaprolactone (PCL) Fibers

**DOI:** 10.3390/polym15142997

**Published:** 2023-07-10

**Authors:** Addison Faglie, Rachel Emerine, Shih-Feng Chou

**Affiliations:** Department of Mechanical Engineering, College of Engineering, The University of Texas at Tyler, Tyler, TX 75799, USA

**Keywords:** electrospun fibers, poloxamers, drug release, mechanical properties, wound dressings

## Abstract

Electrospun microfibers are emerging as one of the advanced wound dressing materials for acute and/or chronic wounds, especially with their ability to carry drugs and excipients at a high loading while being able to deliver them in a controlled manner. Various attempts were made to include excipients in electrospun microfibers as wound dressing materials, and one of them is poloxamer, an amphiphilic polymer that exhibits wound debridement characteristics. In this study, we formulated two types of poloxamers (i.e., P188 and P338) at 30% (*w*/*w*) loading into electrospun polycaprolactone (PCL) fibers to evaluate their physicomechanical properties, biocompatibility, and in vitro drug release of a model drug. Our findings showed that the incorporation of poloxamers in the PCL solutions during electrospinning resulted in a greater “whipping” process for a larger fiber deposition area. These fibers were mechanically stiffer and stronger, but less ductile as compared to the PCL control fibers. The incorporation of poloxamers into electrospun PCL fibers reduced the surface hydrophobicity of fibers according to our water contact angle studies and in vitro degradation studies. The fibers’ mechanical properties returned to those of the PCL control groups after “dumping” the poloxamers. Moreover, poloxamer-loaded PCL fibers accelerated the in vitro release of the model drug due to surface wettability. These poloxamer-loaded PCL fibers were biocompatible, as validated by MTT assays using A549 cells. Overall, we demonstrated the ability to achieve a high loading of poloxamers in electrospun fibers for wound dressing applications. This work provided the basic scientific understanding of materials science and bioengineering with an emphasis on the engineering applications of advanced wound dressings.

## 1. Introduction

Poloxamer is an amphiphilic polymer that belongs to a unique class of synthetic tri-block copolymers, consisting of a central non-polar polypropylene oxide (PPO) core and polar polyethylene oxide (PEO) end caps. This tri-block structure, which simultaneously contains hydrophilic and hydrophobic segments, results in unique properties such as thermosensitivity for use as a mucoadhesive in in situ gels [1], as well as micelle formation for drug delivery applications [2]. Some of the chemical characteristics of poloxamers, such as temperature-dependent self-assembly and thermoreversible behavior, along with biocompatibility, mild inflammatory nature, and non-cytotoxicity, enable poloxamer-based biomaterials to become promising candidates for biomedical application [3]. Poloxamer also provides great versatility with the microstructure, bioactivity, and mechanical properties of the tissue scaffolds, which can be tailored to mimic the behavior of various types of tissues. For example, it was shown that the mechanical properties of nanofibers were improved with the addition of poloxamer [4]. The addition of a copolymer resulted in a much more porous nanofiber structure, and the thermal stability of the nanofibers were improved.

Poloxamers are generally abbreviated by a letter “P” followed by a three-digit number. In particular, the first two digits provide the approximate molecular mass of the PPO core after multiplying by 100. The last digit informs the percentage of PEO end caps’ content after multiplying by 10. The properties and performance of poloxamers can be modified by altering their chain length, the molecular weight of the PPG block, and the PEG percentage [5]. One of the properties of poloxamers is associated with the surfactant nature that enables them to be widely used in drug delivery and wound healing applications [6,7]. In addition, studies showed that hydrogels made from poloxamers were able to retain moisture to provide a suitable environment for the healing of chronic wounds [8]. Recently, poloxamers were incorporated in wound care devices (e.g., UCS^®^, medi UK Ltd., Hereford, UK) to enable wound debridement functions [9]. During debridement, poloxamers break down the interface between water and oils and/or bacteria, so their hydrophobic cores are able to surround the dead tissues, which forms a micelle structure with hydrophilic surfaces for relative ease of cleaning. As a result of their excellent ability in wound debridement and cleaning, poloxamers were investigated in several studies for wound cleaning, biofilm management, and antimicrobial efficacy [7,10,11].

Current topical wound dressings are gel- and ointment-based products, where frequent cleaning and reapplication of the dressing become a burden on both patients and caregivers. Compared to traditional off-the-shelf gel- and ointment-based wound dressings, electrospun fibers possess advantages in (1) high-drug loading (up to 60% *w*/*w*) and encapsulation efficiency (up to 100%); (2) polymer diversity to accommodate physicochemically distinct agents; (3) ability to modulate release; and (4) process simplicity and cost-effectiveness [12]. Electrospun fibers, with diameters ranged from several hundreds of nanometers to a few micrometers, are emerging as the ideal substrate for topical drug delivery and tissue engineering applications [13]. The setup for electrospinning involves the use of a power supply that provides a strong electric field to charge the polymer solution into a jet form to reach the collector. Typical parameters in electrospinning include the applied voltage, the flow rate of the polymer solution, and the distance between the two electrodes.

Several studies incorporated poloxamers in electrospun fibers for biomedical applications. For example, studies showed that poloxamer 407 and polyethylene oxide (PEO) were electrospun into fibers to improve the dissolution and bioavailability of a poorly soluble drug, carvedilol [14]. Others formulated and characterized poly(ε-caprolactone-*co*-lactide)/poloxamer fibers for potential applications in skin tissue engineering [4]. In addition, poloxamers were blended with biopolymers (dextran, gelatin, and agarose) as well as polyacrylonitrile (PAN) to investigate if the polymer solutions were electrospinnable or resulted in electrospraying [15]. Similar work was reported on the blending of 6.79% of poloxamer with 7.5% of PAN, and the results showed electrospraying with a thick structured coating on the substrate fibers [16]. Finally, others used poloxamer to modify the surface properties of an electrospun PU/PCL tube’s luminal surface to enhance the hydrophilicity and bioactive properties [17].

Herein, we electrospun microfibers from polycaprolactone (PCL) with the incorporation of poloxamer 188 (P188) and poloxamer 338 (P338) for potential applications in wound dressing materials. We examined the physicomechanical properties and the in vitro biocompatibilities of the electrospun poloxamer-incorporated PCL fibers. In addition, we evaluated the effects of these poloxamers on the in vitro release behaviors of a small molecule. We demonstrated that electrospun fibers with a high loading of poloxamers (i.e., 30% *w*/*w*) maintained a similar fibrous network. In addition, the mechanical properties of the PCL fibers were related to the incorporation of the poloxamers. Furthermore, results from the dissolution study showed weight losses due to the dissolution of poloxamers, a key mechanism to promote wound debridement functions using poloxamer-incorporated fiber dressings. Finally, we investigated the in vitro biocompatibility of the electrospun poloxamer-incorporated PCL fibers with A549 cells, as well as the in vitro release of a small molecule drug after the incorporation of poloxamers in electrospun PCL fibers.

## 2. Material and Methods

### 2.1. Materials

Polycaprolactone (PCL) with an average molecular weight (M_w_) of 80 kDa, was purchased from Huaian Ruanke Trade Co., Ltd. (Huaian City, China). Poloxamer 188 (P188) and poloxamer 338 (P338) were kindly supplied by BASF (Florham Park, NJ, USA). Ibuprofen (IBP) (98% purity) was purchased from Ark Pharm Inc. (Arlington Height, IL, USA). Hexafluoro-2-propanol (HFIP) was purchased from Richest Group Ltd. (Shanghai, China). Phosphate-buffered saline (PBS) buffer solution (pH ~7.4) was purchased from Avantor (Radnor, PA, USA). All the other chemicals were of reagent grade and used as received without further purification.

### 2.2. Preparation of Polymer Solutions

A total of 15 wt% (*w*/*v*) of PCL solution was prepared by mixing PCL in HFIP solvent in glass vials, followed by placing them on a Thermo Scientific Labquake^TM^ (Waltham, MA, USA) rotisserie mixer overnight at room temperature. After dissolution of PCL, a pre-determined amount of poloxamer powders were added to the PCL solution at 30% *w*/*w* loading (poloxamer/overall polymer), followed by continuous mixing for an hour. Each solution was visually examined carefully for undissolved particles prior to electrospinning.

### 2.3. Electrospinning of PCL/Poloxamer Fibers

All PCL/poloxamer fibers were electrospun within 48 h of the solution preparation. Prior to electrospinning, polymer solution was drawn into a 3 mL Luer-Lok^TM^ BD syringe (Franklin Lakes, NJ, USA) that was attached to a 21-gauge blunt needle. The syringe and needle assembly were then placed onto a NE-1000 programmable single syringe pump (New Era Pump Systems Inc., Farmingdale, NY, USA) to deliver polymer solution at a flow rate of 25 μL/min. During electrospinning, the applied voltage was set at 10 kV over a distance of 10 cm between the tip of the needle and the grounded stationary collector covered with a layer of wax paper. A total of 2 mL of the polymer solution was electrospun from all polymer solutions, recorded by the programmable syringe pump, which was regularly calibrated. After each electrospinning, fiber meshes were covered with aluminum foils and stored in a vacuum desiccator to further remove excess solvents before fiber characterizations.

### 2.4. Fiber Morphology and Diameters

The morphologies of electrospun PCL, PCL/P188, and PCL/P338 fibers were evaluated by a scanning electron microscope (SEM). Briefly, circular disc punches obtained from the fiber mesh were sputter-coated with an Au/Pd target for 30 s using a SPI sputter-coater (West Chester, PA, USA) between 80–100 mTorr. SEM micrographs were acquired from an Aspex tungsten filament SEM system (Delmont, PA, USA) at 15 kV, using a spot size of three, and a working distance of 10.0 mm.

Fiber diameters were measured using ImageJ software (Version 1.53t, National Institutes of Health, Bethesda, MD, USA) on the collected SEM images. In particular, 30 random measurements were taken from different fibers in the SEM images to determine the average fibers diameter and the corresponding standard deviations of each sample (n = 30).

### 2.5. Fiber Deposition Area Measurements

PCL/poloxamer fiber mat thickness measurements were performed by using a digital thickness gauge (resolution = 10 μm). Briefly, a 15 cm × 15 cm grid paper was used to map the locations for the thickness measurements on PCL/poloxamer fiber mats. Each thickness measurement was collected at nodes, 1 cm apart from each other.

Fiber mat thickness data from grid measurements at all nodes were then transferred to MATLAB (Natick, MA, USA) to generate 2D temperature plots to inform the 3D topographical data on thickness distribution. The 2D temperature plots were then imported to ImageJ with fixed settings on the histograms of various sample plots to determine the deposition area of the fiber mats (n = 3).

### 2.6. Fourier Transformed Infrared Spectroscopy (FTIR)

FTIR analyses were carried out by taking electrospun PCL/poloxamer fiber samples and placing them on a Nicolet iS10 ATR-FTIR spectrophotometer from Thermo Fisher Scientific (Walthem, MA, USA). The infrared spectra were collected between wavenumbers ranging from 650 cm^−1^ to 4000 cm^−1^, using a resolution of 8 cm^−1^.

### 2.7. Water Contact Angle Measurements

Surface wettability of the fiber mesh was determined using static water-contact angle measurements on the blank PCL and PCL/poloxamer fiber mats. Briefly, a 4-µL PBS droplet was dispensed on a dry fiber disc at room temperature. An image was taken immediately after the placement of the PBS droplet for static measurement, where the contact angle was measured by ImageJ (Version 1.53t, National Institutes of Health, Bethesda, MD, USA) using the angle between the base surface and the contact tangent of the water droplet (n = 5).

Water absorption rate of the fiber mesh was determined using dynamic water contact angle measurement. Briefly, a video recording was taken during the placement and absorption event of the 4-µL PBS droplet for each PCL/poloxamer fiber disc. Images at various recording frames were selected for contact angle measurements on a sample (resolution = 0.01 s). In particular, the starting time (t = 0 s) was determined when the droplet was initially stabilized on the fiber disc, whereas images of various frames were selected for analysis of dynamic contact angle measurements over a period of time (n = 5).

### 2.8. Fiber Degradation Assays

Fiber degradation assays associated with poloxamer dissolutions were carried using weight measurements from the fiber samples. Briefly, ¾” diameter circular disks were taken from the PCL/poloxamer fiber mats using a metal die. Prior to the degradation assays, initial weights of PCL/poloxamer fiber discs (W_i_) were measured by a Metter Toledo AG245 analytical balance (Columbus, OH, USA). The disc samples were then placed into glass vials containing pre-determined amount of PBS pre-warmed at 37 °C, and incubated at the same temperature using a Thermo Scientific^TM^ MaxQ 4450 orbital shaker (Waltham, MA, USA) at 200 rpm for degradation study.

At the pre-determined time points, fiber discs were removed from their individual vials and dried overnight at room temperature. Sample masses were measured daily until reaching an equilibrium mass, denoted as the final weight (*W*_f_). Poloxamer dissolution rates were determined from the weight changes in the fiber disc samples, as indicated in the equation below (n = 3).

$$\%Weight\;Change=\left(\frac{W_{i}-W_{f}}{W_{i}}\right)\times 100\%$$

### 2.9. Mechanical Tests

Dog-bone tensile specimens of 22 mm in nominal length and 5 mm in width, based on the previous work [18], were carefully prepared by punching the electrospun fiber mats using an ODC stainless steel die (Waterloo, ON, Canada), according to ASTM standard D1708-18 [19]. The thickness of each sample at the nominal region was measured by a digital thickness gauge (resolution = 10 μm).

Uniaxial tensile tests were performed on a single column screw-driven Instron^®^ 3342 universal materials testing machine (Norwood, MA, USA), equipped with a 100 N load cell under 24 ± 1 °C and 45 ± 5% RH, in accordance with ASTM standard D5034-21 [20]. The applied strain rate was 0.01/s on the samples. Load and displacement data were recorded from the instrument for calculation of the stress–strain curve of each sample. Young’s modulus (slope of the initial linear region), tensile strength (zero slope or the highest stress), and elongation to failure (fracture strain) were determined from the corresponding stress–strain curve of each sample in Microsoft Excel (Office 365, Redmond, WA, USA) (n = 5).

### 2.10. In Vitro Biocompatibility Assays

Human lung epithelial cell line (A549) was obtained from the American Type Culture Collection (ATCC) (Manassas, VA, USA). The cells were maintained in Minimal Essential Medium/Nutrient Mixture F12 Ham (MEM/F12 1:1 mixture) medium supplemented with 10% fetal bovine serum (FBS) and 1% antibiotic mixture, and incubated in a humidified chamber with 5% CO_2_ at 37 °C. For biocompatibility assays, the cells were sub-cultured in 12-well tissue culture plates with 100,000/well, and incubated for 2–3 days or until reaching confluency.

Confluent monolayers were exposed to treatment carriers of DMEM/F12 from Thermo Fisher Scientific (Waltham, MA, USA) as a negative control, PBS solution exposing to PCL blank fibers for 2 days, 40 µg/mL concentration of poloxamer/PBS solutions, and PBS solution exposing to PCL/poloxamer fibers for 2 days at 37 °C. The treatments were then replaced with fresh DMEM/F12 medium (500 µL/well) before the addition of 10 µL/well of MTT reagent. The cells were returned to incubator for 4 h at 37 °C with a 5% CO_2_ gas mixture. Metabolically active cells converted the tetrazole of the MTT reagent to purple formazan crystals. A 100 µL of the warm detergent solution was then added to each well, gently mixed, and left for two hours at room temperature in the dark. The supernatants were mixed gently and transferred to a 96-well plate for optical measurements using a Beckman Coulter AD340 plate reader (Brea, CA, USA) at 570 nm absorbance. The percentage of viable cells treated with the polymer extracts was calculated by referring to the carrier-treated control samples with assumed 100% viable cells (n = 5).

### 2.11. In Vitro Drug Release Assays

IBP-incorporated (15% *w*/*w*) PCL and PCL/poloxamer fibers were electrospun using the same parameters as described above. Circular disks with a diameter of 5/8″ were taken from each electrospun IBP-loaded PCL, PCL/P188, and PCL/P338 fiber mesh using a metal die. Masses of the fiber discs were measured by a Metter Toledo AG245 (Columbus, OH, USA) analytical balance. Based upon mass of each disc, a pre-determined amount of PBS solutions were prepared as the release media and pre-warmed to 37 °C in a Thermo Scientific^TM^ MaxQ 4450 (Waltham, MA, USA) orbital shaker at 200 rpm. IBP-loaded fiber samples were then placed in the corresponding vials in the incubator shaker for in vitro release of IBP in the sink condition. At predetermined time points of 0.5, 1, 2, and 4 h, a 40-µL sample of the release media, containing an unknown concentration of the IBP, was removed from the corresponding vial and placed in a 200-µL microcentrifuge tube for UV-vis analysis.

Standard solutions of IBP at concentrations of 400, 200, 100, 50, and 10 µg/mL were prepared using serial dilution methods. Liquid specimens of the standard and unknown IBP samples, collected at various time points, were analyzed using a Thermo Scientific NanoDrop^TM^ 1000 UV-Vis spectrophotometer (Waltham, MA, USA) at 265 nm. The resulting intensities of IBP at 265 nm from the unknown samples were compared to the standard IBP curves to determine the IBP concentrations of the collected liquid samples at each time point. Cumulative release of IBP was calculated using ratios of IBP concentration at various time points and the theoretical concentration of the IBP-loaded fibers in the release media. Results were averaged on three independent measurements (n = 3).

### 2.12. Statistical Analyses 

Results were expressed as average ± standard deviation (SD). Statistical studies of the averages were performed using GraphPad Prism (San Diego, CA, USA) on one-way analysis of variance (ANOVA). Significance was accepted with *p* < 0.05.

## 3. Results and Discussion

### 3.1. Solution Preparation and Fiber Electrospinning

The initial observation on the solution behaviors of poloxamers in PCL solutions at 10% *w*/*w* loading suggested the miscibility of the polymers and the solvent. After the successful initial trial, poloxamer loadings were increased to 30% *w*/*w* in PCL solutions. After thorough mixing, no particles and/or residuals were observed, and the polymer mixtures became clear and viscose solutions ideal for electrospinning.

**Figure 1:**
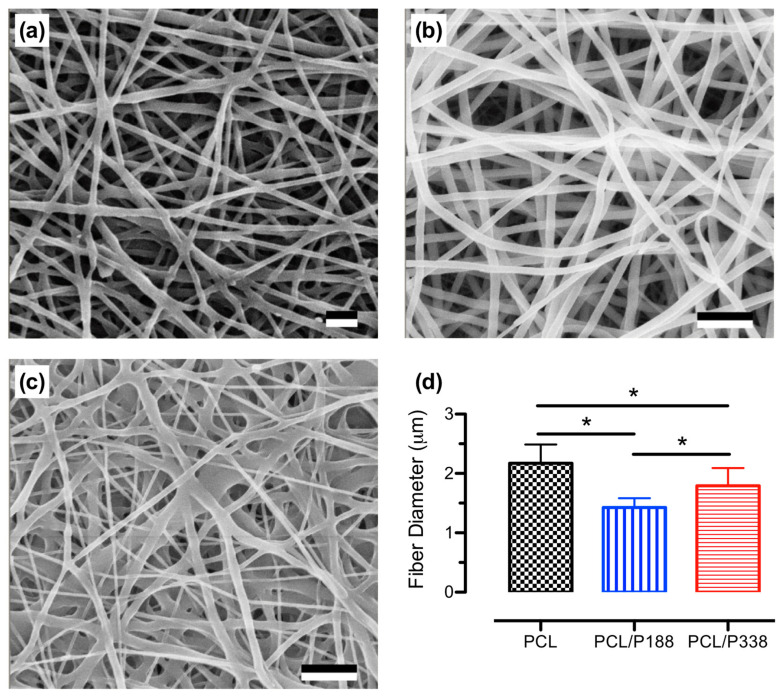
Representative SEM images of electrospun fibers, showing fiber morphologies of (**a**) PCL control, (**b**) PCL/P188, and (**c**) PCL/P338 groups. Scale bar = 10 μm. (**d**) Average fiber diameters of blank PCL, PCL/P188, and PCL/P338 groups (n = 30). An asterisk between the groups indicates statistical significance (*p* < 0.05).

After electrospinning, disc samples of PCL control, PCL/P188, and PCL/P338 fibers were prepared for SEM imaging on their corresponding fiber morphologies and microstructures. Representative SEM images of the electrospun PCL/poloxamer fibers are shown in Figure 1a–c for PCL control, PCL/P188, and PCL/P338 fibers, respectively. According to the observations, PCL/poloxamer fibers showed smooth fiber surfaces and a uniform fiber mat structure without defects, similar to those from the PCL control groups. These observations suggested that P188 and P338 were electrospinnable with PCL at 30% *w*/*w* loading. In a similar study, loading of poloxamers (e.g., 10% and 33%) on fiber morphologies of PCL were reported, where the fiber mats showed interconnected porous structures at 10% poloxamer loading [4]. Increasing poloxamer loading to 33% increased the levels of fiber fusing at the cross points. This defective microstructure, typically associated with slow solvent evaporation during electrospinning, was not observed in our PCL/P188 and/or PCL/P338 fibers at 30% *w*/*w* loading.

SEM images of PCL control, PCL/P188, and PCL/P338 fibers were analyzed by ImageJ software to obtain the average fiber diameters as shown in Figure 1d. The average fiber diameters for PCL control, PCL/P188, and PCL/P338 groups were statistically different at 2.17 ± 0.32 μm, 1.43 ± 0.16 μm, and 1.79 ± 0.30 μm, respectively (*p* < 0.05). According to the data, incorporation of poloxamers into PCL fibers resulted in the decrease in average fiber diameters, perhaps due to the improved electrospinnability, resulting in a stronger “whipping” behavior that provided a greater stretching and thinning process to the fibers [21]. Other electrospun blend fibers from polylactic acid (PLA) and regenerated silk fibroin (RSF), with the incorporation of a small molecule drug (i.e., Aspirin, up to 1.1% *w*/*w*), showed a smaller average fiber diameter due to the improvement of the charge density on the surface of the jet flow [22]. Furthermore, comparisons of the average fiber diameters between the PCL/poloxamer groups suggested that increasing molecular weight of the poloxamers increased the average PCL/poloxamer fiber diameters slightly. The average molecular weights of P188 and P338 were approximately 8500 Da and 15,000 Da, respectively. Studies have shown that average fiber diameters increased with polymers having higher average molecular weights [23]. In addition, our electrospun poloxamer-loaded PCL fibers had a smaller average fiber diameter than the commercially available wound dressings, which ranged from 11.5 μm to 20.7 μm [24]. A fibrous wound dressing with a smaller fiber diameter received a higher surface area to volume ratio, where the surface properties of the fibers will dominate the bulk properties of the polymers.

### 3.2. Fiber Deposition Area Measurements

Single-nozzle uniaxial electrospinning of polymer fibers, using a stationary collector, resulted in a cone-shaped deposition of nonwoven fiber meshes. The deposition area of the fibers provided valuable information on the understanding of how the fibers were deposited on the collector plate, as well as the electrospinnability of the polymer solutions [25]. Through the measurements on fiber mat thickness at fixed locations, it was possible to obtain the average fiber deposition area and thickness distributions of fiber meshes that informed the “whipping” behaviors of the fibers and their spreading ability associated with the degree of chain entanglement at the molecular level during electrospinning.

**Figure 2:**
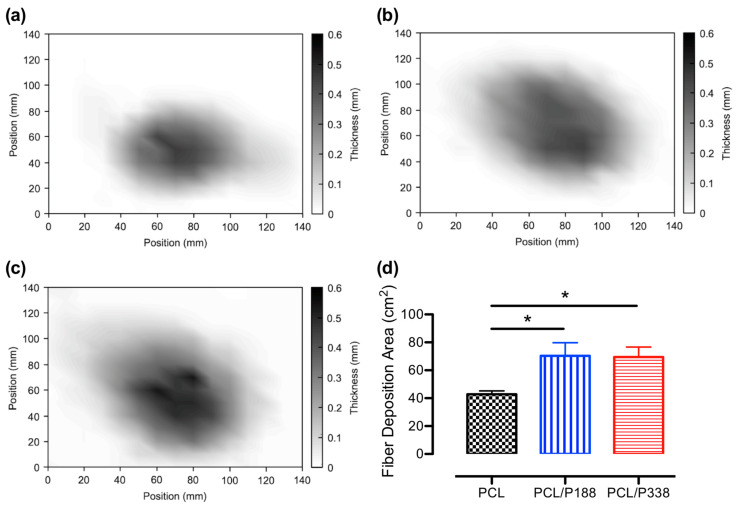
Representative temperature plots on fiber thickness of (**a**) PCL control, (**b**) PCL/P188, and (**c**) PCL/P338 groups. Scales are in mm. (**d**) Average fiber deposition areas of PCL control, PCL/P188, and PCL/P338 groups (n = 3). An asterisk between the groups indicates statistical significance (*p* < 0.05).

Fiber mesh thickness measurements were performed using a digital thickness gauge on a grid paper aligned with the collector plate to ensure the exact locations of the measurements between fiber meshes. Representative temperature plots on thickness distributions of PCL control, PCL/P188, and PCL/P338 fiber meshes are shown in Figure 2a–c, respectively. PCL control fibers showed a smaller and more concentrated fiber deposition area than those of the poloxamer-loaded PCL fibers. Specifically, the PCL control samples displayed a rounded fiber mat deposition area, whereas the poloxamer-loaded PCL fibers exhibited some levels of diagonally stretched elliptical edge, suggesting an increased level of fiber “whipping” during electrospinning. The average fiber distribution areas are shown in Figure 2d, where the average fiber deposition areas of PCL/P188 and PCL/P338 fibers were 70.3 ± 9.4 cm^2^ and 69.5 ± 7.2 cm^2^, respectively. These values were significantly larger than that of the blank PCL fibers (42.8 ± 2.4 cm^2^) (*p* < 0.05).

Studies showed that gravity played a role in deposition area of the electrospun TiO_2_/PVP fibers, where increasing the angles between the electric field and the gravity increased the average grain size of the TiO_2_, but decreased the average fiber diameter [26]. At a higher angle, the droplet changed shape and size, resulting in a higher charge density on the surface of the solution jet during electrospinning. Since the PCL control and PCL/poloxamer fibers were electrospun with the same setup configurations and conditions, the addition of the poloxamers not only enhanced the chain entanglement but also improved the charge density of the droplets. The latter effect promoted the concentration of electric stresses at the jet tip, resulting in higher protraction forces on the droplets [27]. The improvement on charge density of the solution jet led to extensive plastic deformation during the fiber forming process in electrospinning, which was known as an unstable and whipping motion of the jet termed bending instability [28]. In general, our data suggested that the poloxamer-loaded PCL fibers produced a much larger average fiber deposition area than the PCL control fibers due to enhanced “whipping” behaviors during electrospinning, as a result of poloxamers’ ability to improve chain entanglement and charge density of the polymer solution.

### 3.3. FTIR

FTIR studies were performed on the PCL control, PCL/P188 and PCL/P338 fibers to confirm the incorporation and encapsulation of poloxamers within PCL fibers. Poloxamers can be identified as a tri-block copolymer, where the two end terminals of the central hydrophobic polypropylene oxide (PPO) molecular chain are attached to hydrophilic polyethylene oxide (PEO) molecular chains. The PEO consists of ethylene groups (–CH_2_CH_2_–)_n_ at the carbon backbone linked by ether linkages (–O–). Similarly, PPO exhibits the same carbon backbone structure, where one of the hydrogens on the ethylene group is replaced with a methyl group (–CH_3_). In contrast, PCL contains ester linkage (–COO–) at the carbon backbone.

**Figure 3:**
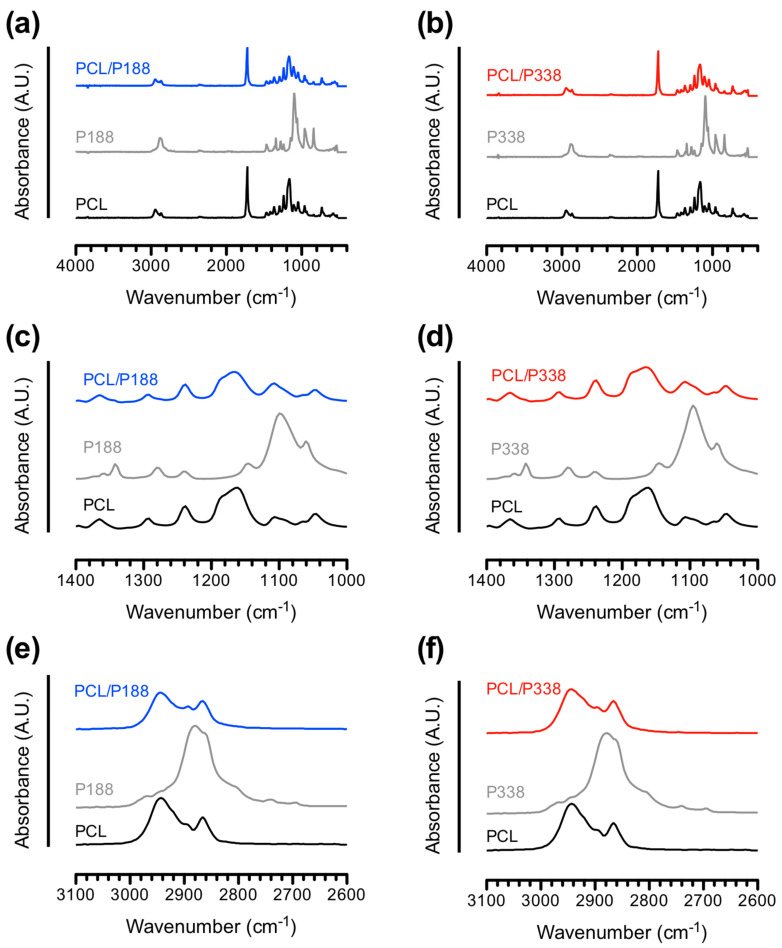
FTIR spectra of (**a**) PCL/P188 and (**b**) PCL/P338 fibers in comparison with PCL control fibers and poloxamer raw powders from 650 cm^−1^ to 4000 cm^−1^, (**c**) PCL/P188 and (**d**) PCL/P338 fibers in comparison with PCL control fibers and poloxamer raw powders in the finger print region, (**e**) PCL/P188 and (**f**) PCL/P338 fibers in comparison with PCL control fibers and poloxamer raw powders in the high-wavenumber region.

Figure 3a–f shows the FTIR spectra of PCL/P188 and PCL/P338 fibers, respectively, as compared to the spectra of PCL control fibers and the corresponding raw poloxamer powders. The most distinct peak from the poloxamers was at a wavenumber of 1100 cm^−1^ associated with C–O–C symmetric stretching vibration [17,29], where a small peak at 1150 cm^−1^ overlapping with the broad band of C–O–C indicated its asymmetric stretching vibration [30]. In addition, characteristic peaks at 960 cm^−1^ and 845 cm^−1^ corresponded to CH_2_ rocking/CO stretching and –CH_2_–CO– rocking/stretching on the carbon backbone of poloxamers, respectively [31]. Other noticeable characteristic peaks of poloxamers included symmetric stretching of –CH_2_ and wagging/twisting of –CH_2_ bands at wavelengths 2883 cm^−1^ and around 1345 cm^−1^, respectively [32]. Others investigated the chemical structure of poloxamers using FTIR and suggested the similar observations from our measurements [33,34].

The FTIR spectra of PCL included –C=O stretching vibrations of the ester carbonyl group at 1727 cm^−1^, which represented the strongest peak in the entire spectrum [35]. The symmetric and asymmetric stretching vibrations of C–O–C from the ester linkages in the carbon backbone of PCL were also noticeable in the range of 1100 cm^−1^ and 1150 cm^−1^. In addition, bands at 2943 cm^−1^ and 2883 cm^−1^ corresponded to –C–H asymmetric stretching and –C–H symmetric stretching [36]. The C–O–C stretching vibration and –C–H symmetric stretching bands of PCL overlapped with those from the poloxamers. In general, the PCL/P188 and PCL/P338 spectra showed the combined characteristic bands of PCL and poloxamers with no additional peaks and/or peak shifts, suggesting the successful incorporation and encapsulation of the poloxamer in PCL with minimal secondary force interactions (e.g., hydrogen bonds) between the two polymers [37].

### 3.4. Surface Wettability and Water Absorption Rates

Poloxamers have been reported as potential candidates to improve the dissolution and release rates of poorly water-soluble drugs [6]. In this study, PCL fibers were electrospun as a platform for topical drug delivery applications, based on our previous work in modulating drug release rates through drug-polymer interactions [18]. However, PCL is a hydrophobic polymer and electrospun PCL fiber mats exhibit micro- to nano-sized porous architectures that impede the wettability of the fiber mats due to the high-surface tension of the biological fluids. Therefore, the incorporation of poloxamers in electrospun PCL fibers may provide an alternative solution in modulating drug release rates associated with surface wettability of the drug carriers.

**Figure 4:**
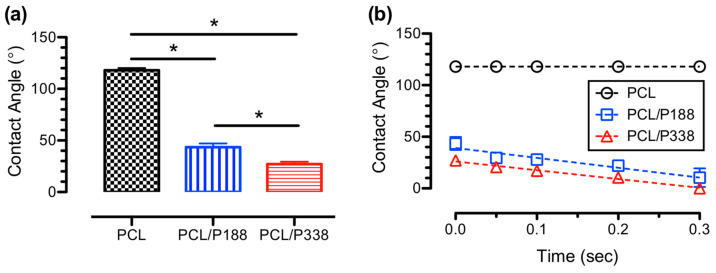
(**a**) Average surface wettability of the PCL control, PCL/P188, and PCL/P338 fiber meshes using static water contact angle measurements (n = 5). An asterisk between the groups indicates statistical significance (*p* < 0.05). (**b**) Average water absorption rates of the blank PCL, PCL/P188, and PCL/P338 fiber meshes using dynamic water contact angle measurements (n = 5). Dashed lines represent linear fitting of the data.

Surface wettabilities of the fiber meshes were determined from average water contact angle measurements. The results are shown in Figure 4a, and the average water contact angle from PCL fiber mats was 118.0° ± 3.8°, whereas the average water contact angles for PCL/P188 and PCL/P338 fiber mats were significantly lower at 43.5° ± 6.3° and 27.1° ± 3.9°, respectively (*p* < 0.05). These findings were in accordance with others, where P338 was added as a coating to silicon urinary catheters to decrease the average water contact angles from 95° ± 5° (silicon controls) to 72° ± 3° (silicon/P338), in order to reduce biofilm formation [38]. Others showed a decrease in average water contact angles from 70° ± 2° (controls) to 12° ± 1° (poloxamer groups) after incorporating poloxamers in electrospun silk fibroin scaffolds [39].

During the water contact angle measurements, PCL/P188 and PCL/P338 fiber mats continuously absorbed the water droplets over time. As a result, water absorption rates were determined from dynamic water contact angle measurements, as shown in Figure 4b. Results suggested that the PCL/P188 and PCL/P338 fibers mats exhibited a linear decrease in water contact angles over time. The slopes for the PCL/P188 and PCL/P338 groups were −95.7°/s and −85.3°/s, respectively. Our results were significantly greater than the reported values from the literature due to the differences between porous membranes and solid films [40,41]. In another study, dynamic water contact angle measurements using surfactants were measured on porous sponges, where various stages of surface wetting mechanisms were suggested [42]. The rate changes on the water contact angle, depending on the types of sponges used and the initial degree of water saturation on the sponges, ranged from 1.2°/s to 16.5°/s. The reported data were several-folds slower than the current electrospun PCL/poloxamer fiber mats, suggesting the importance of the pore size differences (e.g., 93~300 μm from the sponges) on the rate of water absorption. In general, time-dependent water contact angle measurements provided an alternative method to evaluate the water uptake ability in electrospun fibers.

### 3.5. Fiber Degradation Studies

Poloxamers have shown great wound debridement abilities when incorporated into gel/cream dressings [11]. However, poloxamer loading in gels/creams was limited by its critical micelle concentrations (CMC) [43]. To enable a high concentration of poloxamer in drug carriers as solid dosages, electrospun fibers appear to the one of the best candidates. After incorporating 30% *w*/*w* poloxamers in electrospun PCL fibers, we performed in vitro degradation studies to measure the average weight changes in the fibers, which indirectly inform the release of poloxamers from PCL/poloxamer fibers.

**Figure 5:**
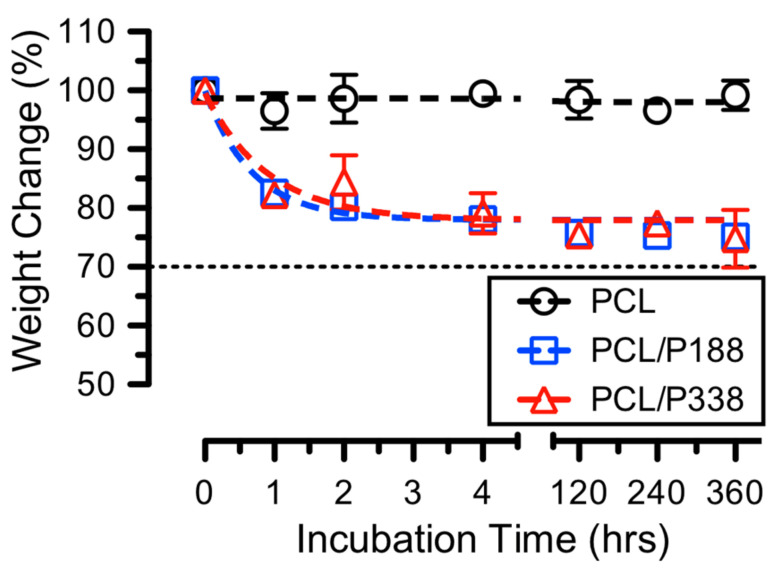
Average weight changes in PCL control, PCL/P188, and PCL/P338 fibers in PBS incubated at 37 °C for up to 15 days (n = 3). Dashed lines represented one phase association fitting of the data. The 70% dashed line referenced the loading of poloxamer.

The average percentage weight changes in PCL/P188 and PCL/P338 fibers over time are shown in Figure 5. Since PCL required several years to fully degrade (t_1/2_ > 18 months) [44], it was anticipated that the average weight changes measured were primarily associated with dissolution of poloxamer in the PBS. In particular, PCL control fibers showed minimal weight changes (<3%) during the study (i.e., 15 days). For PCL/P188 and PCL/P338 fibers, results indicated average weight changes of 82.6 ± 1.7% and 82.2 ± 0.7% of the original weight after 1 h of incubation, respectively. The average weight changes in the PCL/P188 and PCL/P338 fibers continued to decrease to 78.1 ± 1.5% and 79.1 ± 3.4% of the original weights after 4 h of incubation, respectively. The rate constants (K), using one phase association curve fitting, suggested values of 2.2 and 1.9 from PCL/P188 and PCL/P338 fibers, respectively. These weight change values maintained to the end of the incubation time of 15 days for the investigation of this study. These findings indicated that poloxamers were capable of dissolving and/or diffusing out from PCL/poloxamer fibers within several hours.

### 3.6. Mechanical Properties

**Figure 6:**
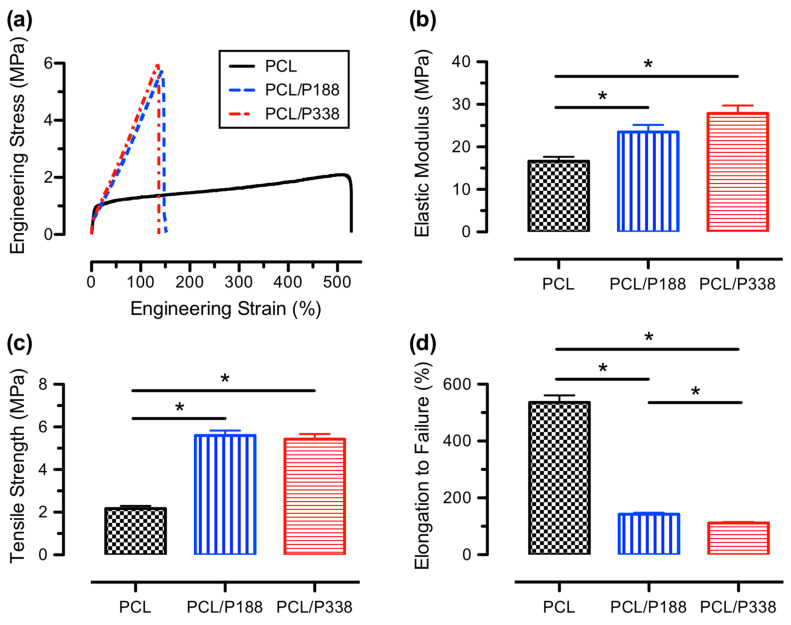
Mechanical properties of PCL control, PCL/P188, and PCL/P338 fibers, showing (**a**) representative stress–strain curves, (**b**) average elastic moduli, (**c**) average tensile strengths, and (**d**) average elongation to failure (n = 5). An asterisk between the groups indicates statistical significance (*p* < 0.05).

Uniaxial tensile tests were performed on the electrospun blank PCL and PCL/poloxamer fibers, and the representative engineering stress–strain curves are shown in Figure 6a. The stress–strain curves exhibited an initial linear viscoelastic region, followed by a yielding behavior where stresses increased minimally, especially for the blank PCL groups with increasing strains, similar to our previous findings [45,46]. The yielding behavior was associated with the permanent deformation on the PCL backbones due to the unfolding and stretching of the amorphous regions. This process was further limited due to the incorporation of poloxamers as a result of molecular chain entanglements between poloxamers and PCL. As strains continued to increase, permanent deformation occurred within PCL semi-crystalline chain conformations. Such behavior was associated with a slight increase in stress when increasing strain, known as the strain-hardening region, prior to fracture. Comparing to semi-crystalline polyurethane films, the stress–strain curves of the electrospun PCL and PCL/poloxamer fibers showed similar mechanical behaviors of another semicrystalline polymer [47].

The average elastic moduli of the blank PCL, PCL/P188 and PCL/P338 fibers are shown in Figure 6b. The incorporation of poloxamers in PCL fibers significantly increased the average moduli from 16.6 ± 1.8 MPa to 23.5 ± 2.9 MPa and 27.9 ± 3.2 MPa for blank PCL, PCL/P188 and PCL/P338 fibers, respectively (*p* < 0.05). The increases in average elastic moduli were associated with the incorporations of poloxamers in PCL fibers, perhaps due to improvements of molecular chain entanglement resulting in a stiffer chain response upon stretching. Our findings were in accordance with a study using a small molecule drug (i.e., cilostazol) up to 18.75% in electrospun PCL fibers, where the increases in average moduli were related to the intermolecular and intramolecular drug-polymer interactions [48]. In addition, the incorporation of a higher molecular weight of poloxamer (e.g., P338) in the PCL fibers increased the average elastic moduli of the fibers.

The average tensile strengths, shown in Figure 6c, were 2.2 ± 0.2 MPa, 5.6 ± 0.4 MPa, and 5.4 ± 0.4 MPa for blank PCL, PCL/P188, and PCL/P338 fibers, respectively. These values were somewhat lower than the reported data [4]; however, they were similar to our previous reported value for the blank PCL [18]. The poloxamer-load PCL fibers exhibited significant higher average tensile strengths than that of the blank PCL fibers (*p* < 0.05). The increase in average tensile strength of PCL/poloxamer fibers was associated with molecular chain entanglement during electrospinning that further improved the intermolecular interactions to stabilize the PCL backbones. This finding was further supported by others where the average tensile strengths of PCL fibers depended on the degree of crystallinity and molecular orientation [49,50].

The average elongation to failure, shown in Figure 6d, of the PCL/P188 and PCL/P338 fibers decreased to 142 ± 9% and 112 ± 7%, respectively, from that of the blank PCL fibers of 536 ± 43%. All groups exhibited statistical significance as compared with others (*p* < 0.05). The decreases in average elongation to failure for the PCL/poloxamer fibers were correlated to the increase in the average tensile strength, where the intermolecular interactions, associated with the chain entanglement, played an important role on a more glassy-typed fracture [51].

In a recent study, 11 frequently used commercial wound dressings were studied and their physicochemical properties were reported [24]. These wound dressings include passive gauze and tulle, interactive dressings, such as films, foams, and hydrogels, and bioactive dressings, such as hydrocolloids, hydrofibers, and alginates. In particular, 9 out of the 11 commercial wound dressings investigated had the elastic moduli lower than 1 MPa. The Carbonet^◊^ wound dressing consisted of fibrous cellulose pad and an adsorbing activated charcoal layer sandwiched between the polyethylene nets, where the elastic modulus was around 6 MPa. Furthermore, the only comparable commercial wound dressing was the Acticoat^◊^ with an elastic modulus of a little over 35 MPa, which consisted of an absorbent rayon/polyester layer sandwiched between two antimicrobial silver-coated polyethylene nets. The highest tensile strength of these commercial wound dressings was lower than 5 MPa (i.e., Acticoat^◊^). However, three of the investigated commercial wound dressings had a total elongation of more than 700%. In general, the mechanical properties of our poloxamer-loaded PCL fibers stand close with these commercial wound dressings, or even better considering their elastic modulus and tensile strength.

**Figure 7:**
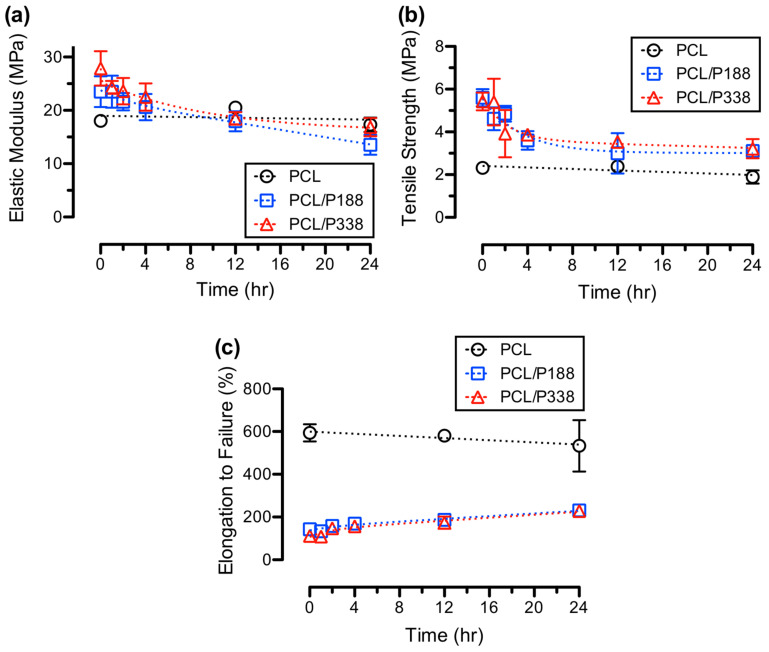
Mechanical properties of PCL control, PCL/P188, and PCL/P338 fibers after incubating in PBS for various hours, showing (**a**) average elastic moduli over time, (**b**) average tensile strengths over time, and (**c**) average elongation to failure over time (n = 5).

Electrospun PCL control and PCL/poloxamer fibers were mechanically stretched after various incubation times to investigate the changes in mechanical properties after poloxamers dissolution. The average elastic moduli, shown in Figure 7a, of the PCL/P188 and PCL/P338 fibers before incubation were 23.5 ± 2.9 MPa and 27.9 ± 3.2 MPa, respectively. After 1 h of incubation in PBS, the corresponding average elastic moduli decreased to 21.6 ± 1.6 MPa and 20.1 ± 4.6 MPa for PCL/P188 and PCL/P338 fibers, respectively. After 24 h of incubation, the average elastic moduli of the fibers further decreased to values that were comparable to the PCL control fibers (16.6 ± 1.8 MPa).

Similar to average elastic moduli, the average tensile strength, shown in Figure 7b, were found to decrease with increasing incubation time reaching to the average tensile strength of the PCL control fibers. The average tensile strength of the PCL/P188 and PCL/P338 fibers before incubation were 5.6 ± 0.4 MPa and 5.4 ± 0.4 MPa, respectively. After 24 h of incubation, the average tensile strength decreased to 3.1 ± 0.2 MPa and 3.2 ± 0.5 MPa for the PCL/P188 and PCL/P338 fibers, respectively. The average tensile strength of the PCL control fibers slightly decreased from 2.2 ± 0.2 MPa to 1.9 ± 0.3 MPa.

The average elongation to failure, shown in Figure 7c, of the PCL/poloxamer fibers increased with incubation time, while the average elongation to failure of the PCL control fibers slightly decreased after 24 h of incubation. The average elongation to failure was 536 ± 120%, 231 ± 17%, and 228 ± 11% for PCL control, PCL/P188, and PCL/P338 fibers after 24 h of incubation, respectively. The ductility of the PCL/poloxamer fibers only received a minimal increase on the average elongation to failure, suggesting the possibility of large macroscopic defects in fiber networks at a large deformation associated with poloxamer leaving the polymer matrix.

### 3.7. In Vitro Biocompatibility Assays

**Figure 8:**
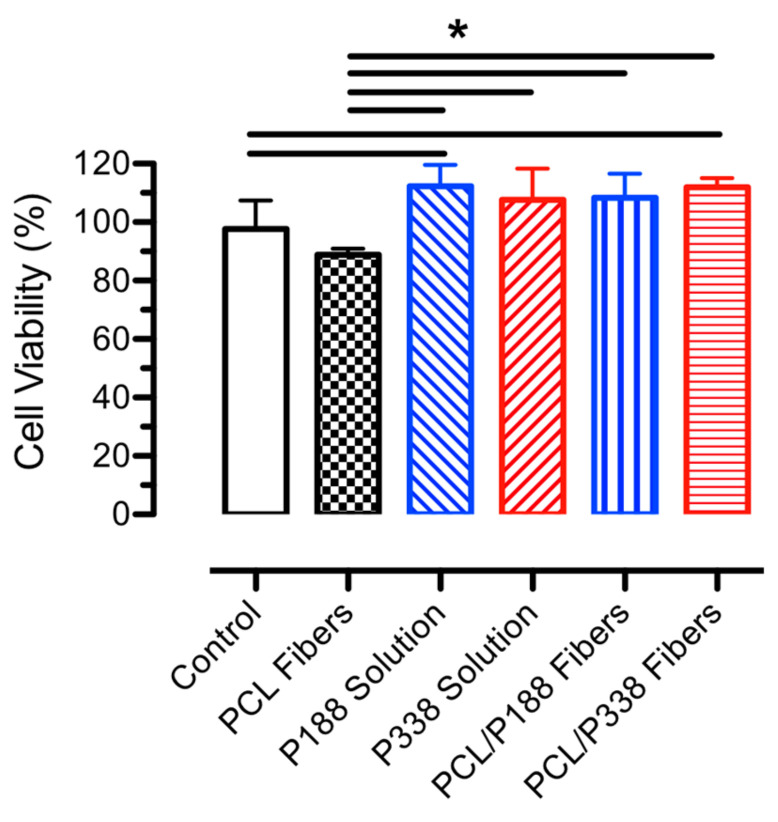
Viabilities of human lung epithelial cells with PCL control, PCL/P188, and PCL/P338 fibers along with other control groups over 48 h of culture (n = 5). An asterisk between the groups indicates statistical significance (*p* < 0.05).

The viabilities of human lung epithelial cells were evaluated by MTT assays in the presence of PCL control and PCL/poloxamer fibers, as shown in Figure 8. Mitochondrial functioning of the living cells, as indicated by the UV absorption of formazan forming crystals, showed minimal cytotoxicities after 48 h of exposure to both PCL control and PCL/poloxamer fibers, as compared to the control groups, including those of poloxamer solutions. Cell viabilities ranged from 88.9 ± 2.1% (PCL control groups) to 112.2 ± 7.4% (P188 solution groups), where the PCL fiber groups were statistically different than P188 solutions, P338 solutions, PCL/P188 fiber, and PCL/P388 fiber groups (*p* < 0.05). Our results showed that electrospun PCL/poloxamer fibers were compatible with human lung epithelial cells within 48 h. PCL is known for its biocompatibility and has been widely used in many biomedical applications [52]. For example, studies showed that PCL fibers have low toxicity toward mouse fibroblasts (3T3) over 3 days, with a two-fold increase in cell counts over 14 days [53]. Others produced lipid drug carriers containing poloxamer 407, and the viability of the human lung cancer cells (A549) slightly decreased to 90% when the poloxamer 407 concentration was increased to 0.8 mg/mL [54]. This reported concentration is 20 times greater than our poloxamer concentrations (i.e., 40 µg/mL) in the cell viability assay, which explained the low toxicity finding in the results. Moreover, studies showed that nanoparticles made from PCL/Pluronic F68 had minimal effects in viability of the A549 cells [55]. Overall, our electrospun PCL/poloxamer fibers demonstrated excellent biocompatibility to cell cultures, which can be served as a drug delivery platform for wound healing applications.

### 3.8. In Vitro Drug Release Assays

To further understand the effects of poloxamer-incorporated electrospun PCL fibers on drug delivery, an in vitro drug release assay in the sink condition was performed using ibuprofen (IBP) as a model drug. Through the single-nozzle uniaxial electrospinning, IBP was homogeneously distributed from the core to the outer surface of the fibers. As a result, the surface drug will typically provide a burst release followed by the diffusion of the core drug through the polymer matrix [56]. Specifically, wetting at the fiber surfaces promoted drug transportation in the fibers, where poloxamer incorporated PCL fibers showed a higher affinity to wetting, compared to the PCL controlled fibers (Figure 4).

**Figure 9:**
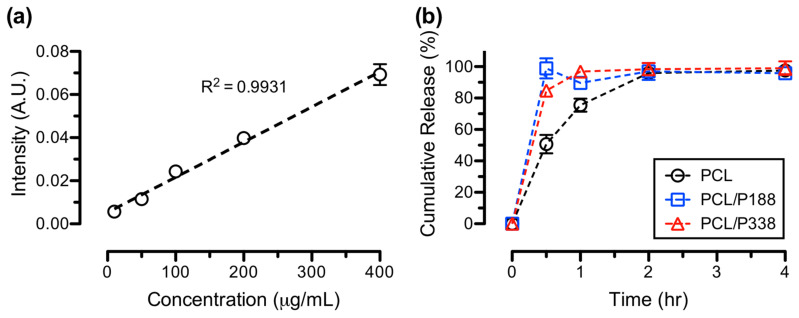
In vitro IBP release studies, showing (**a**) IBP standard curve from 10 µg/mL to 400 µg/mL and (**b**) IBP release profiles from PCL control, PCL/P188, and PCL/P338 fibers (n = 3).

The in vitro release curves of IBP from the PCL control, PCL/P188, and PCL/P338 fibers are shown in Figure 9. After 0.5 h, the cumulative releases of IBP were 50.7 ± 5.8%, 98.9 ± 6.4%, and 84.8 ± 2.5% for PCL control, PCL/P188, and PCL/P338 fibers, respectively. Both PCL/P188 and PCL/P338 fibers reached complete release of IBP at 0.5 h time point and the IBP concentrations remained the same through 4 h of incubation in PBS. In contrast, cumulative IBP release gradually increased to 75.5 ± 4.1% and 95.9 ± 0.9% at 1 h and 2 h time points for PCL control fibers, respectively. Studies demonstrated the fast release characteristics of small molecule drugs after incorporation of poloxamer 407 in electrospun hydrophilic fibers, due to the surface wetting and improvement in dissolution of poorly water-soluble drugs [14,57]. In general, poloxamer promoted the wettability of hydrophobic PCL fibers to facilitate the release of small molecule model drugs.

## 4. Conclusions

In conclusion, we formulated two types of poloxamers in PCL fibers at a loading of 30% (*w*/*w*) for potential applications in wound dressing materials. Our findings showed that poloxamer-incorporated PCL fibers had smooth and defect free microstructure, where the fiber mechanical properties became less ductile with an improvement in stiffness and strength. In addition, incorporating poloxamer in the PCL fibers reduced the hydrophobicity, and the dissolution studies showed that poloxamers were able to leach out from the PCL fiber over 4 h. These findings on the surface properties of the poloxamer-incorporated PCL fibers supported the results from the in vitro drug release studies that the model small-molecule drug had a faster initial release as compared to the PCL controlled fibers. Finally, poloxamer-incorporated PCL fibers were biocompatible with A549 cells. Overall, our studies showed that poloxamers are an excellent excipient in electrospun fibers, and perhaps other types of tissue scaffolds, that can be used as wound dressing materials.

## Data Availability

All data included in this study are available upon request by contacting the corresponding author.

## References

[1] Hirun N., Kraisit P., Tantishaiyakul V. (2022). Thermosensitive polymer blend composed of poloxamer 407, poloxamer 188 and polycarbophil for the use as mucoadhesive in situ gel. Polymers.

[2] Oh K.T., Bronich T.K., Kabanov A.V. (2004). Micellar formulations for drug delivery based on mixtures of hydrophobic and hydrophilic Pluronic^®^ block copolymers. J. Control. Release.

[3] Zarrintaj P., Ramsey J.D., Samadi A., Atoufi Z., Yazdi M.K., Ganjali M.R., Amirabad L.M., Zangene E., Farokhi M., Formela K. (2020). Poloxamer: A versatile tri-block copolymer for biomedical applications. Acta Biomater..

[4] Pan J., Liu N., Sun H., Xu F. (2014). Preparation and characterization of electrospun PLCL/poloxamer nanofibers and dextran/gelatin hydrogels for skin tissue engineering. PLoS ONE.

[5] Dun J., Osei-Yeboah F., Boulas P., Lin Y., Sun C.C. (2020). A systematic evaluation of poloxamers as tablet lubricants. Int. J. Pharm..

[6] Bodratti A., Alexandridis P. (2018). Formulation of poloxamers for drug delivery. J. Funct. Biomater..

[7] Das Ghatak P., Mathew-Steiner S.S., Pandey P., Roy S., Sen C.K. (2018). A surfactant polymer dressing potentiates antimicrobial efficacy in biofilm disruption. Sci. Rep..

[8] Du L., Tong L., Jin Y., Jia J., Liu Y., Su C., Yu S., Li X. (2012). A multifunctional in situ-forming hydrogel for wound healing: In situ-forming hydrogel for wound healing. Wound Repair Regen..

[9] Khatun S. (2016). Demystifying debridement and wound cleansing. J. Community Nurs..

[10] Percival S.L., Mayer D., Malone M., Swanson T., Gibson D., Schultz G. (2017). Surfactants and their role in wound cleansing and biofilm management. J. Wound Care.

[11] Percival S.L., Chen R., Mayer D., Salisbury A.-M. (2018). Mode of action of poloxamer-based surfactants in wound care and efficacy on biofilms. Int. Wound J..

[12] Chou S.-F., Carson D., Woodrow K.A. (2015). Current strategies for sustaining drug release from electrospun nanofibers. J. Control. Release.

[13] Iacob A.-T., Drăgan M., Ionescu O.-M., Profire L., Ficai A., Andronescu E., Confederat L.G., Lupașcu D. (2020). An overview of biopolymeric electrospun nanofibers based on polysaccharides for wound healing management. Pharmaceutics.

[14] Kajdič S., Vrečer F., Kocbek P. (2018). Preparation of poloxamer-based nanofibers for enhanced dissolution of carvedilol. Eur. J. Pharm. Sci..

[15] Böttjer R., Grothe T., Wehlage D., Ehrmann A. (2018). Electrospraying poloxamer/(bio-)polymer blends using a needleless electrospinning machine. J. Text. Fibrous Mater..

[16] Wehlage D., Böttjer R., Grothe T., Ehrmann A. (2018). Faculty of Engineering and Mathematics, Bielefeld University of Applied Sciences, 33619 Bielefeld, Germany Electrospinning water-soluble/insoluble polymer blends. AIMS Mater. Sci..

[17] Le A.N.-M., Tran N.M.-P., Phan T.B., Tran P.A., Tran L.D., Nguyen T.H. (2021). Poloxamer additive as luminal surface modification to modulate wettability and bioactivities of small-diameter polyurethane/polycaprolactone electrospun hollow tube for vascular prosthesis applications. Mater. Today Commun..

[18] Chou S.-F., Woodrow K.A. (2017). Relationships between mechanical properties and drug release from electrospun fibers of PCL and PLGA blends. J. Mech. Behav. Biomed. Mater..

[19] (2018). Standard Test Method for Tensile Properties of Plastics by Use of Microtensile Specimens.

[20] (2021). Standard Test Method for Breaking Strength and Elongation of Textile Fabrics (Grab Test).

[21] Sarabi-Mianeji S., Scott J., Pagé D.J.Y.S. (2015). Impact of electrospinning process parameters on the measured current and fiber diameter. Polym. Eng. Sci..

[22] Qin J., Jiang Y., Fu J., Wan Y., Yang R., Gao W., Wang H. (2013). Evaluation of drug release property and blood compatibility of aspirin-loaded electrospun PLA/RSF composite nanofibers. Iran. Polym. J..

[23] Beachley V., Wen X. (2009). Effect of electrospinning parameters on the nanofiber diameter and length. Mater. Sci. Eng. C.

[24] Minsart M., Van Vlierberghe S., Dubruel P., Mignon A. (2022). Commercial wound dressings for the treatment of exuding wounds: An in-depth physico-chemical comparative study. Burns Trauma.

[25] Zargham S., Bazgir S., Tavakoli A., Rashidi A.S., Damerchely R. (2012). The effect of flow rate on morphology and deposition area of electrospun nylon 6 nanofiber. J. Eng. Fibers Fabr..

[26] Al-Hazeem N.Z., Ahmed N.M., Mat Jafri M.Z., Ramizy A. (2021). The effect of deposition angle on morphology and diameter of electrospun TiO_2_ /PVP nanofibers. Nanocomposites.

[27] Stanger J., Tucker N., Kirwan K., Staiger M.P. (2009). Effect of charge density on the Taylor cone in electrospinning. Int. J. Mod. Phys. B.

[28] Kiselev P., Rosell-Llompart J. (2012). Highly aligned electrospun nanofibers by elimination of the whipping motion. J. Appl. Polym. Sci..

[29] Garala K., Joshi P., Patel J., Ramkishan A., Shah M. (2013). Formulation and evaluation of periodontal in situ gel. Int. J. Pharm. Investig..

[30] Obaidat R.M., AlTaani B., Ailabouni A. (2017). Effect of different polymeric dispersions on In-vitro dissolution rate and stability of celecoxib class II drug. J. Polym. Res..

[31] Bergeron C., Perrier E., Potier A., Delmas G. (2012). A study of the deformation, network, and aging of polyethylene oxide films by infrared spectroscopy and calorimetric measurements. Int. J. Spectrosc..

[32] Vasconcelos T., Prezotti F., Araújo F., Lopes C., Loureiro A., Marques S., Sarmento B. (2021). Third-generation solid dispersion combining Soluplus and poloxamer 407 enhances the oral bioavailability of resveratrol. Int. J. Pharm..

[33] Vyas V., Sancheti P., Karekar P., Shah M., Pore Y. (2009). Physicochemical characterization of solid dispersion systems of tadalafil with poloxamer 407. Acta Pharm..

[34] Dhillon R., Ojha R., Bedi N. (2014). Preparation, characterization and optimization of poloxamer solid dispersions of a poorly water soluble drug aprepitant. Br. J. Pharm. Res..

[35] Jia Y., Huang G., Dong F., Liu Q., Nie W. (2016). Preparation and characterization of electrospun poly(ε-caprolactone)/poly(vinyl pyrrolidone) nanofiber composites containing silver particles. Polym. Compos..

[36] Elzein T., Nasser-Eddine M., Delaite C., Bistac S., Dumas P. (2004). FTIR study of polycaprolactone chain organization at interfaces. J. Colloid Interface Sci..

[37] Tran T.T.D., Tran P.H.L. (2020). Molecular interactions in solid dispersions of poorly water-soluble drugs. Pharmaceutics.

[38] Stirpe M., Brugnoli B., Donelli G., Francolini I., Vuotto C. (2020). Poloxamer 338 affects cell adhesion and biofilm formation in *Escherichia coli*: Potential applications in the management of catheter-associated urinary tract infections. Pathogens.

[39] Kadakia P.U., Growney Kalaf E.A., Dunn A.J., Shornick L.P., Sell S.A. (2018). Comparison of silk fibroin electrospun scaffolds with poloxamer and honey additives for burn wound applications. J. Bioact. Compat. Polym..

[40] Wang X., Chen Z., Shen Z. (2005). Dynamic behavior of polymer surface and the time dependence of contact angle. Sci. China Ser. B.

[41] Goswami S., Klaus S., Benziger J. (2008). Wetting and absorption of water drops on nafion films. Langmuir.

[42] Johnson P., Routledge T., Trybala A., Vaccaro M., Starov V. (2019). Wetting and spreading of commercially available aqueous surfactants on porous materials. Colloids Interfaces.

[43] Suksiriworapong J., Rungvimolsin T., A-gomol A., Junyaprasert V.B., Chantasart D. (2014). Development and characterization of lyophilized diazepam-loaded polymeric micelles. AAPS PharmSciTech.

[44] Peña J., Corrales T., Izquierdo-Barba I., Doadrio A.L., Vallet-Regí M. (2006). Long term degradation of poly(ε-caprolactone) films in biologically related fluids. Polym. Degrad. Stab..

[45] Chou S.F., Overfelt R.A. (2011). Tensile deformation and failure of North American porcupine quills. Mater. Sci. Eng. C.

[46] Chou S.F., Overfelt R.A., Miller M.E. (2012). Anisotropic mechanical behavior of keratin tissue from quill shells of North American porcupine (*Erethizon dorsatum*). Mater. Sci. Eng. A.

[47] Wilson A.C., Chou S.-F., Lozano R., Chen J.Y., Neuenschwander P.F. (2019). Thermal and physico-mechanical characterizations of thromboresistant polyurethane films. Bioengineering.

[48] Rychter M., Baranowska-Korczyc A., Milanowski B., Jarek M., Maciejewska B.M., Coy E.L., Lulek J. (2018). Cilostazol-loaded poly(ε-caprolactone) electrospun drug delivery system for cardiovascular applications. Pharm. Res..

[49] Wong S.-C., Baji A., Leng S. (2008). Effect of fiber diameter on tensile properties of electrospun poly(ɛ-caprolactone). Polymer.

[50] Selli F., Erdoğan U.H., Hufenus R., Perret E. (2020). Mesophase in melt-spun poly(ϵ-caprolactone) filaments: Structure–mechanical property relationship. Polymer.

[51] Ahmad R., Paul S., Basu S. (2020). Characterization of entanglements in glassy polymeric ensembles using the Gaussian linking number. Phys. Rev. E.

[52] Azari A., Golchin A., Mahmoodinia Maymand M., Mansouri F., Ardeshirylajimi A. (2021). Electrospun polycaprolactone nanofibers: Current research and applications in biomedical application. Adv. Pharm. Bull..

[53] Moyers-Montoya E.D., Escobedo-González R.G., Vargas-Requena C.L., Garcia-Casillas P.E., Martínez-Pérez C.A. (2021). Epithelial growth factor-anchored on polycaprolactone/6-deoxy-6-amino-β-cyclodextrin nanofibers: In vitro and in vivo evaluation. Polymers.

[54] Hajipour H., Nouri M., Ghorbani M., Bahramifar A., Emameh R.Z., Taheri R.A. (2021). Targeted nanostructured lipid carrier containing galangin as a promising adjuvant for improving cytotoxic effects of chemotherapeutic agents. Naunyn. Schmiedebergs Arch. Pharmacol..

[55] Patel P., Raval M., Manvar A., Airao V., Bhatt V., Shah P. (2022). Lung cancer targeting efficiency of Silibinin loaded Poly Caprolactone /Pluronic F68 Inhalable nanoparticles: In vitro and In vivo study. PLoS ONE.

[56] Hawkins B.C., Burnett E., Chou S.-F. (2022). Physicomechanical properties and in vitro release behaviors of electrospun ibuprofen-loaded blend PEO/EC fibers. Mater. Today Commun..

[57] Emerine R., Chou S.-F. (2022). Fast delivery of melatonin from electrospun blend polyvinyl alcohol and polyethylene oxide (PVA/PEO) fibers. AIMS Bioeng..

